# Recent progress of eco-friendly manufacturing process of efficient perovskite solar cells

**DOI:** 10.1186/s40580-023-00375-5

**Published:** 2023-06-12

**Authors:** Nayoon Kwon, Jaehee Lee, Min Jae Ko, Young Yun Kim, Jangwon Seo

**Affiliations:** 1 Department of Chemical and Biomolecular Engineering, Korea Advanced Institute of Science and Technology (KAIST), Daejeon, 34141 Republic of Korea; 2 Department of Chemical Engineering, Hanyang University, Seoul, 04763 Republic of Korea; 3 Division of Advanced Materials, Korea Research Institute of Chemical Technology (KRICT), 141 Gajeong-ro, Yuseong-gu, Daejeon, 34114 Republic of Korea

**Keywords:** Perovskite solar cells, Environmentally friendly fabrication, Green solvent systems, Scale-up production, Antisolvent-free fabrication

## Abstract

Perovskite solar cells (PSCs) have the potential to produce solar energy at a low cost, with flexibility, and high power conversion efficiency (PCE). However, there are still challenges to be addressed before mass production of PSCs, such as prevention from degradation under external stresses and the uniform, large-area formation of all layers. Among them, the most challenging aspect of mass production of PSCs is creating a high-quality perovskite layer using environmentally sustainable processes that are compatible with industry standards. In this review, we briefly introduce the recent progresses upon eco-friendly perovskite solutions/antisolvents and film formation processes. The eco-friendly production methods are categorized into two: (1) employing environmentally friendly solvents for perovskite precursor ink/solution, and (2) replacing harmful, volatile antisolvents or even limiting their use during the perovskite film formation process. General considerations and criteria for each category are provided, and detailed examples are presented, specifically focused on the works have done since 2021. In addition, the importance of controlling the crystallization behavior of the perovskite layer is highlighted to develop antisolvent-free perovskite formation methods.

**Graphical Abstract:**

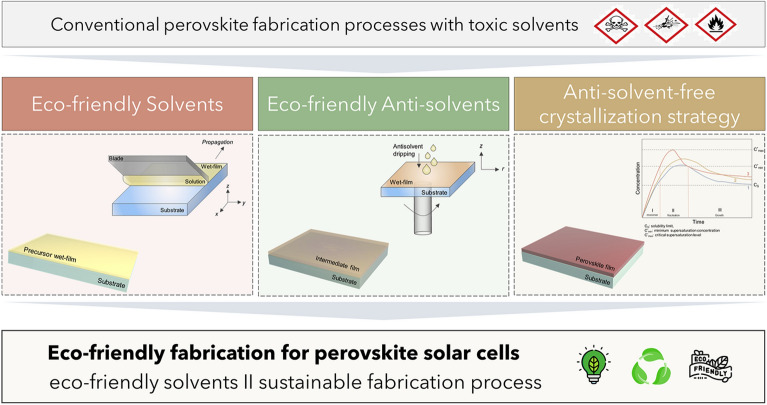

## Introduction

Perovskite solar cells (PSCs) have attracted significant attention because of their superior optoelectronic properties, including high power-conversion efficiency (PCE), flexible form factor, light weight, and potential for low-cost fabrication [1, 2]. The successful demonstration of PSCs with a PCE of over 25% has resulted in not only stronger interest in academic research and development, but also substantial investment by many companies in pilot production lines to mass produce PSCs [3]. However, there are still many obstacles to be overcome before the mass production of PSCs, such as instability under external stresses or the uniform formation of all layers in large areas [4–6].

To mass produce PSCs, the most important but difficult part is the formation of a high-quality perovskite layer with industry-compatible, sustainable processes [6, 7]. Electrodes or charge-transporting layers have already been commercialized for organic/inorganic semiconductor optoelectronic devices and can be easily formed in large areas using a one-step vacuum process or solution processes followed by thermal annealing. However, the perovskite layer exhibits complex crystallization behavior, which requires two separate steps—large-area deposition of a precursor wet-film and complete phase conversion to a photoactive perovskite phase [8, 9]. This makes it difficult to achieve a uniform, high-quality film in large areas. Additionally, the perovskite formation process should be human- and environment-friendly to be applied in mass production lines [10].

A human- and environment-friendly production method for perovskite layers is crucial but has yet to be thoroughly investigated. In particular, the benefit of introducing an eco-friendly solvent system for mass production of PSCs should be systematically investigated. The overall environmental impact of manufacturing processes of PSCs has been studied in several previous works [11, 12]. A large portion of energy and environmental impact is caused by deposition (and patterning) of transparent conducting oxide and high temperature annealing steps, but recycling processes can be a solution to mitigate these impacts. The usage of eco- and human-toxic solvents has also been found to have a profound effect on the long-term environmental impacts [13]. Because the estimated amount of solvent for fabrication of 1 GW PSCs would be 3500 L [14], reducing eco- and human-toxicity derived from those solvent would be crucial. Introduction of an eco-friendly solvent system or ultimately reducing the amount of solvents usage during production would be beneficial not only for reducing health and environmental risks in both short and long run, but also for minimizing the cost from post-treatment and recycling of harmful solvents. In this sense, environmentally friendly production methods can be classified into two categories. The first involves the development of a perovskite precursor ink/solution that only employs environmentally friendly solvents. The second involves substituting the harmful, volatile antisolvent or even limiting the use of antisolvents during the perovskite film formation process.

**Fig. 1:**
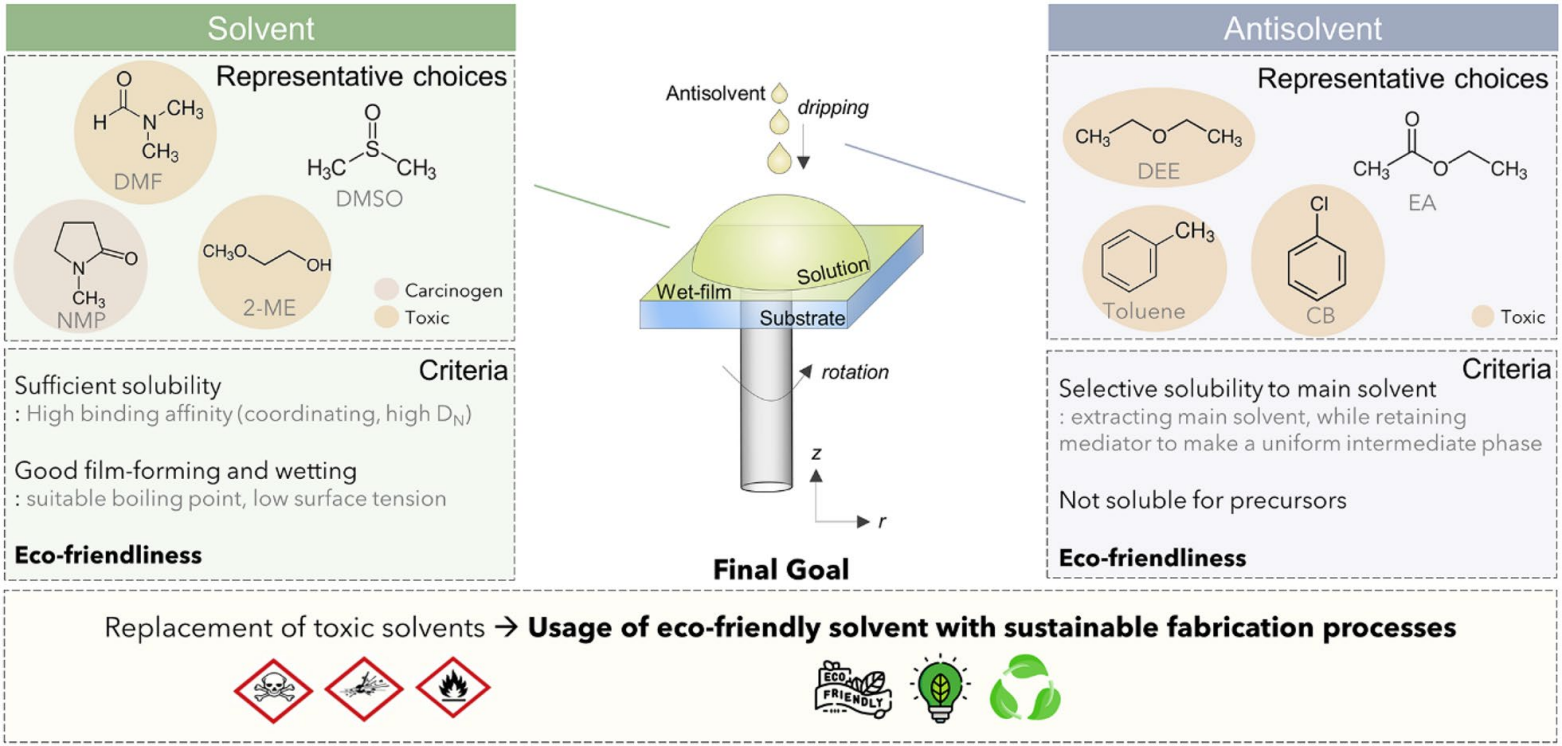
Representative examples and criteria for proper selection of solvents and antisolvents

The selection of solvents for the perovskite precursor ink is limited to polar solvents which have sufficient affinity to the Pb center atom by donating an electron-pair. This is needed to ensure sufficient solubility. The solvent also should be benign for human health and not have adverse effects on the surrounding environment even in the long run. Although polar solvents have a relatively lower risk of dissolution in body fat or of passing through lipid barriers, compared to non-polar solvents, these solvents can be metabolized by enzymes such as alcohol dehydrogenase and aldehyde dehydrogenase to create harmful metabolites or can accumulate to cause long-term damage to organs, and even induce cancer through genomic mutations [15]. Therefore, the selection of an appropriate solvent for mass production is very difficult. Antisolvents have been widely used since the early pioneering work for perovskite film formation [8]. For use in large areas, however, tremendous quantities must be used, compared to the precursor solvents, so they must be chosen while considering their potential harm to human health and the environment, as well as volatility and fire risk. Figure 1 shows the representative solvents and antisolvents typically used in previous works, and the criteria that the solvents/antisolvents should meet.

**Fig. 2:**
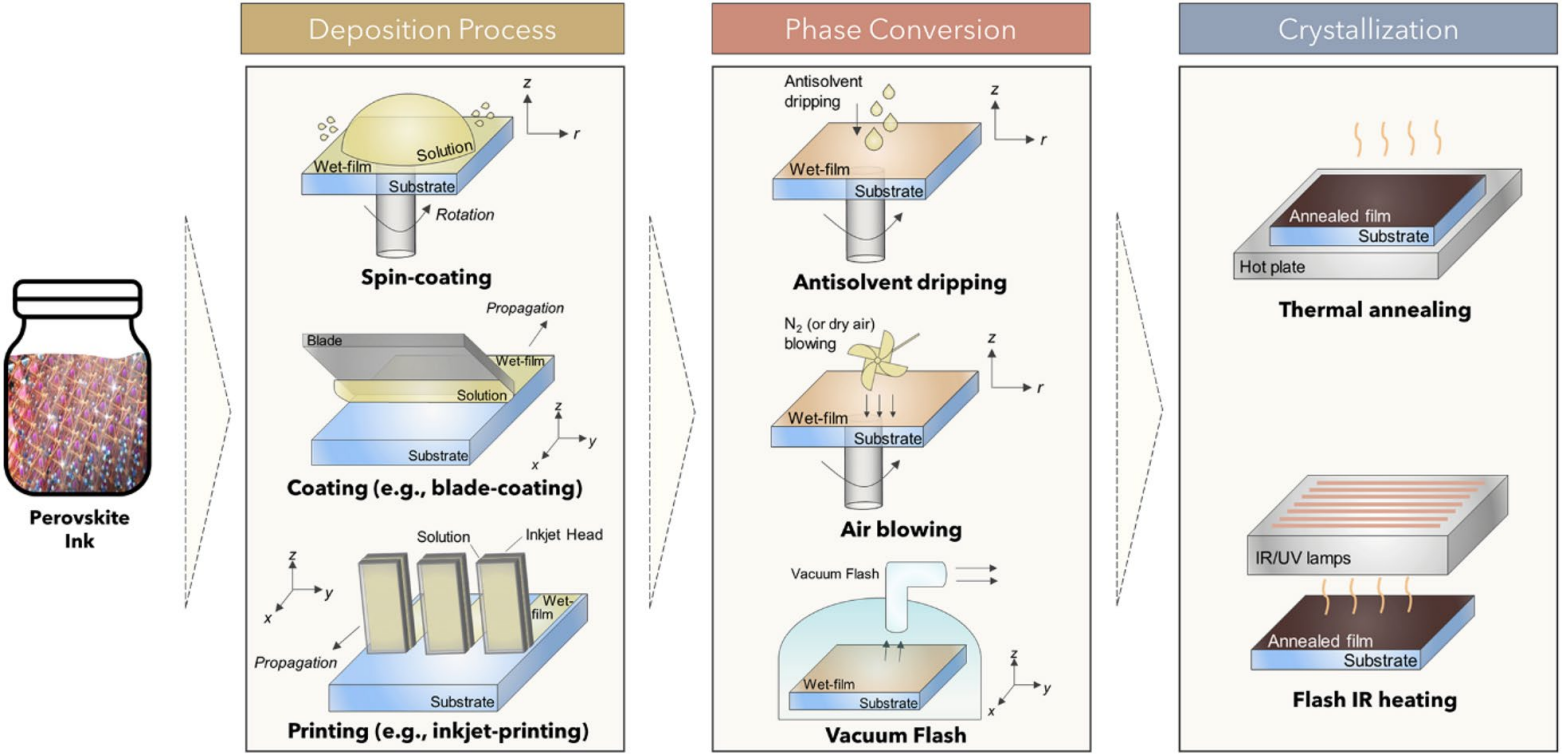
Schematic illustration of perovskite formation processes: perovskite precursor deposition, phase conversion, and crystallization

The perovskite precursor solution, prepared using selected solvents, should ideally include suitable processes such as coating and printing, and allow conversion with a chosen antisolvent from an amorphous precursor phase to a dense, photoactive perovskite phase. (Fig. 2) For large areas, the conventional spin-coating process should be substituted by other scalable processes to reduce wasting large amounts of solvents and to ensure greater uniformity over the entire area. Coating processes including blade coating or printing processes such as inkjet or gravure printing can be utilized to produce large-area films, and have already been demonstrated successfully by many researchers [16–21]. The chosen solvent system should be finely tuned to fit specific scalable processes, because the physical/chemical properties of solvents affect the processability (i.e. wettability, adhesion, interfacial tension, and etc.) of the solution on a desired substrate. A proper phase conversion technique should be applied for new environmentally friendly solvent/antisolvents to guarantee the reliable formation of high-quality film over an entire area. Phase conversion can be easily achieved by conventional antisolvent dripping method, or by applying physical forces to extract main solvent such as vacuum or compressed air blowing. After phase conversion, direct/indirect thermal annealing should be followed to obtain high-quality perovskite film.

Alternatively, a better way to reduce the risk of harmful effects on human health and the environment from such antisolvents, would be to develop a new process that does not require an antisolvent. To accomplish this goal, complete understanding and sophisticated control of the crystallization behavior of the perovskite layer is required. The new process should mimic the pre-nucleation, burst nucleation, and following growth by diffusion processes, using the solvent dripping method to achieve a high-quality, uniform perovskite layer.

In this review, environmentally friendly perovskite processing methods are thoroughly presented, from both perspectives—to substitute solvent/antisolvents with eco-friendly solvents, and to develop antisolvent-free perovskite formation methods. The recent progress in eco-friendly perovskite precursor solutions/antisolvents and film formation processes since 2021 are presented, along with brief explanations and representative works of each technique. The scope of this review does not cover lead leakage prevention [22, 23] or lead-free PSCs [24, 25]. A complete list of eco-friendly solvents can be found in other literature.

## Precursor ink design & solvents for perovskite film fabrication

As mentioned in the 1 section, the solvent for the perovskite precursor should have sufficient solubility while not being harmful to the environment or the human body. Generally, a perovskite precursor solvent is a mixture of a main solvent, co-solvent/mediator, and additives. The main solvent provides sufficient solubility for the precursor to be dissolved completely. For the co-solvent or mediator, a molecule that can form a strong bond to the Pb atoms that comprise the perovskite structure are typically used, to form an intermediate state with the perovskite precursor to prevent uncontrolled, rapid crystallization at room temperature. Additives are introduced separately or together with the co-solvent to improve crystallization behavior and quality. After rapid pouring of the antisolvent, the main solvents are washed away, and the intermediate phase is changed to the perovskite phase by thermal annealing, to evaporate the co-solvent and additive.

There have been some previous reports on solvent screening methods to select proper solvents for perovskite with adequate solubility. Stevenson et al. firstly suggested a new metric, namely, Mayer Bond Order, for prediction of complexation effectiveness in perovskite precursors for various solvent [26]. J. C. Hamil et al. showed that the donor number (DN) is a more effective and accurate predictor than the dielectric constant to determine which solvent should be used for the main solvent and mediator n [27]. Rather than covalent or ionic interactions, solvation of the perovskite precursor is thought to coordinate solvent molecules to the perovskite precursor. Therefore, DN can be an effective criteria for predicting the solubility of various solvents. Generally, solvents with a high DN have a strong binding affinity and a high boiling point, so they are suitable for co-solvents, to maintain an intermediate state at room temperature and induce uniform crystallization behavior during thermal annealing. Recently, rational categorization approach of solvents for perovskite precursor has been proposed by Tutantsev et al. [28] They suggested that combination of three parameters, namely, DN, dipole moment, and solubility parameter for hydrogen bonding should be taken into account for describing the interaction between perovskite precursor and solvent more comprehensively. For example, DN can be a suitable predictor for donor-acceptor interaction between Pb^2+^ ion and solvents, and dipole moment can be used for prediction of solvation of [PbX_n_]^2−n^ complexes by solvent, and solubility parameter for hydrogen bonding also should be considered to predict interaction of solvents with X^−^ and A^+^ ions.

Currently, most of the reported literature on PSCs uses N,N-dimethylformamide (DMF) as the main solvent. DMF has a suitable boiling point and good solubility to the perovskite precursor. However, the use of DMF is restricted in many countries worldwide due to its human toxicities, including its hepatotoxicity and irritations of skin or eyes. As a co-solvent, N,N-dimethylacetamide (DMAc), hexamethylphosphoramide (HMPA) and N-methyl-2-pyrrolidone (NMP) have been used, but they are also severely restricted due to their carcinogenic properties, with the only exception being dimethyl sulfoxide (DMSO). Therefore, a screening for environmentally friendly alternatives is required.

Environmental friendliness should be considered from various aspects or factors, including the lethal dose for 50% kill (LD50), immediate danger to life and health (IDLH), or EU regulations for substances of very high concern (SVHC). Recently, a study on systematically evaluating the human toxicity of perovskite solution using the USEtox method was reported[13]. Such efforts should be made more thoroughly, and any prediction based on a calculation should be carefully matched with experimental toxicological data to prevent unexpected adverse effects from perovskite solvents, and to determine suitable alternatives. The safety information of solvents given in Tables is summarized in Table 1.

**Table 1:** The safety issues and basic properties of various antisolvents

Solvents	Severely harmful statements in GHS^a^	IDLH^b^ [ppm]	LD_50_^c^ [mg/kg]
Limonene	N	–	5200
2-ME	Y	200	1480
2-MeTHF	N	–	300–2000
2-methylpyrazine	N	50	1800
ACN	N	500	2460
Diethyl carbonate	N	–	4900
DMA	Y	300	4300
DMF	Y	500	2800
DMPU	Y	–	1300
DMSO	N	–	14,500
EA	N	2000	5620
EtOH	Y	3300	6000
GBL	N	–	1540
GVL	N	–	8800
IPA	N	2000	5045
Isobutanol	N	1600	3750
MA	N	100	100
MAAc	N	–	-
NMP	N	–	3914
TEP	N	–	500

All the abbreviation for solvents is given in manuscript or following tables

^a^GHS: Globally Harmonized System of Classification and Labelling of Chemicals. Severely harmful statements including H340-341 (genetic defects), H350-351 (cancer), H360-361 (damaging fertility or the unborn child), H362 (harm to breast-fed children), and H370-373 (damaging organs)

^b^IDLH: Immediately Dangerous to Life or Health Values. All the values are obtained from the website of the National Institute for Occupational Safety and Health (NIOSH), United States

^c^LD_50_: Median Lethal Dose. All the values are obtained from the MSDS documents provided by the supplier, for rat, oral

Previous works have mainly focused on replacing DMF with another solvent. Ketones (gamma-butylolactone (GBL), gamma-valerolactone (GVL)), alcohols (ethanol or 2-butoxyethanol (2-BE)), and acetonitrile (ACN) have been reported. Representative reports on the usage of such eco-friendly solvents as main solvents are given below, and recent reports on the usage of DMF-alternative main solvents since 2021 are summarized in Table 2.

**Table 2:** Recent reports on DMF-free fabrication for perovskite films

Main solvent	Structure	Method	PCE%	References
ACN, MA	FTO/c-TiO_2_/mp-TiO_2_/MAPbI_3_/Spiro-OMeTAD/Ag	Solution-shearing	20.30	[29]
TEP	FTO/SnO_2_/(FAPbI_3_)_0.95_(MAPbBr_3_)_0.05_/Spiro-OMeTAD/Au	Spin-coating	20.13	[30]
DMSO, DMPU, 2-MeTHF, EtOH	FTO/SnO_2_/MAPbI_3_/Spiro-OMeTAD/Au	Spin coating	16.1	[31]
DMSO	ITO/PTAA/SiO_2_ NPs/FA0.83Cs0.17Pb(I0.87Br0.13)_3_/PCBM/BCP/Ag	Gas stream assisted blade coating	16.7	[32]
GBL/2-methylpyrazine /DMSO	PET/IZO/PEDOT:PSS/Cs0.1(FA0.83MA0.17)0.9Pb(I0.83Br0.17)_3_/C60/BCP/Ag	Ink-jet printing	11.4	[33]
GVL	FTO/c-TiO_2_/mp-TiO_2_/m-ZrO_2_/m-carbon/AVA0.03MAPbI_3_	Drop casting	12.91	[34]
GVL	FTO/c-TiO_2_/mp-TiO_2_/m-ZrO_2_/m-carbon/AVA0.03MAPbI_3_	Drop casting	13.82	[35]
TEP	FTO/SnO_2_-NRs/(FAPbI3)0.95(MAPbBr3)0.05/Spiro-OMeTAD/Ag	Spin-coating	22.42	[36]
EtOH, DMA	FTO/SnO_2_/FAPbI_3_/Spiro-OMeTAD/Au	Spin-coating	25.1	[37]
2-ME	ITO/MeO-2PACz/MAPbI3-xClx/PC60BM/BCP/Ag	Spin-coating	20.39	[38]
2-ME	FTO/bl-TiO_2_/mp-TiO_2_/(FAPbI_3_)0.95(MAPbBr3)0.05/Spiro-OMeTAD/Au	Air-knife assisted bar-coating	20.3	[39]

Abbreviation: *MA* Methylammonium, *FA* Formamidinium, e.g., *MAPbBr*_*3*_ Methylammonium lead bromide, *FAPbI*_*3*_ Formamidinium lead iodide

**Fig. 3:**
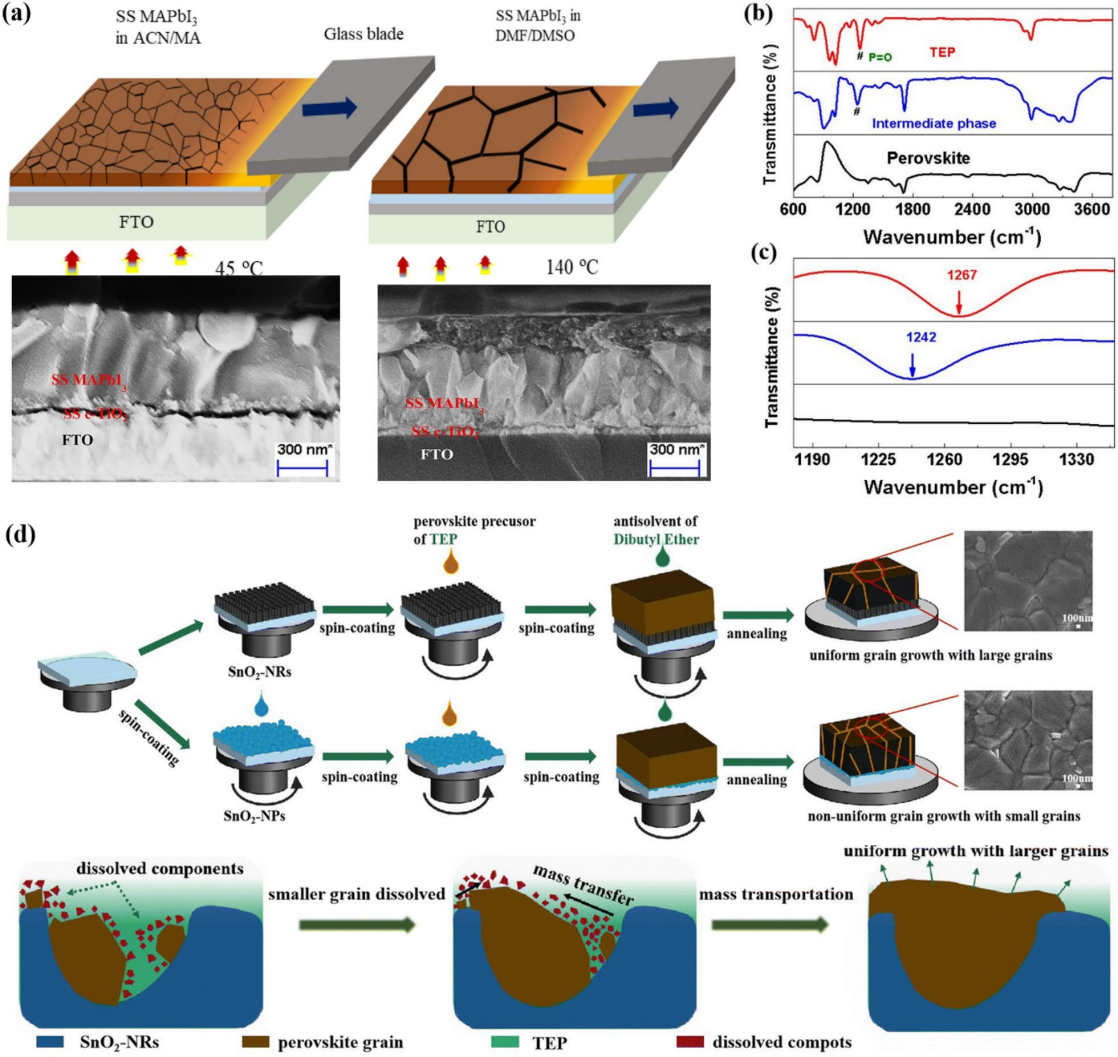
**a **Schematic illustration of a solution-shearing method and cross-sectional Scanning Electron Microscope (SEM) images of perovskite layer on FTO/c-TiO_2_/mp-TiO_2_ substrate prepared at different conditions of DMF/DMSO solution and ACN/MA solution. Reprinted with permission from [29]. Copyright 2022, Elsevier. **b **the full and **c **enlarged FTIR spectra result of TEP, intermediate phase, and perovskite (annealed). Reprinted with permission from [30]. Copyright 2022, Elsevier **d** Schematic of the perovskite layer deposition processes based on ETL substrate. Reprinted with permission from [36]. Copyright 2023, Royal Society of Chemistry

### ACN

ACN has a solubility to perovskite precursors due to polar cyano group, but its limited solubility is an obstacle to be used solely as a main solvent. In previous works, ACN is typically mixed with another co-solvent to improve its solubility. The fumigation of methylamine in gaseous state resulted in an enhancement of solubility via melting of precursors to form a liquid mixture with methylamine[40]. Mixing with tetrahydrofuran (THF) [41] or 2-methoxyethanol (2-ME) [17] is also reported to improve the overall solubility, but toxicity of THF or 2-ME can be also problematic.

In 2022, Belay Adugna et al. [29] employed a solvent engineering strategy to reduce the grain boundaries in solution-sheared (SS) perovskite layers. When using the DMF/DMSO solvent system, which has been the most widely used, it was inevitable to produce films with large boundaries because of the high boiling point of the solvents. Therefore, they used ACN) as the solvent for the perovskite precursor solution to reduce the shearing temperature. For perovskite gel synthesis, pre-synthesized MAPbI_3_ powder was mixed with methylamine (MA) to form a yellow gel. Then, the final solution was prepared by mixing the perovskite gel with ACN in various ratios. The lowered shearing temperature (45℃) successfully improved the film morphology and properties, resulting in a perovskite film with narrow grain boundaries and fewer defects (see Fig. 3a). The fabricated device achieved the PCE of 20.30% with a configuration of fluorine-doped tin oxide (FTO)/c-TiO_2_/mesoporous (mp)-TiO_2_/MAPbI_3_/2,2′,7,7′-tetrakis[N,N-di(4-methoxyphenyl)amino]-9,9′.

-spirobifluorene (Spiro-OMeTAD)/Ag, which is higher than that achieved using the conventional DMF/DMSO solution.

### TEP

**Fig. 4:**
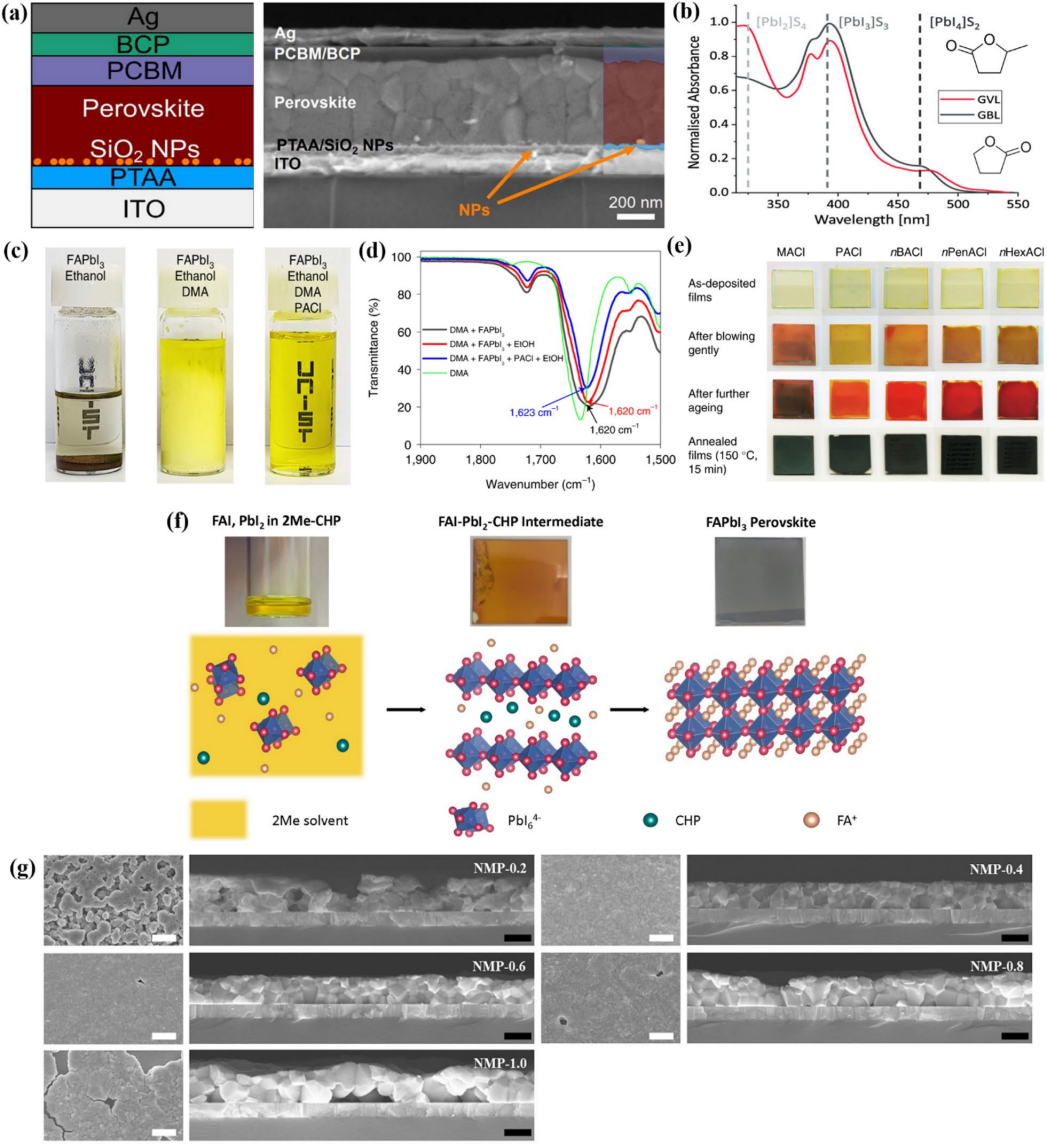
**a** Schematic illustration of device architecture and cross-sectional SEM image of a PSC fabricated from the DMSO solution system. Reprinted with permission from [32], Copyright 2021, American Chemical Society. **b** UV—vis spectra of perovskite precursors in GVL and GBL. Reprinted with permission from [34], Copyright 2021, The Authors. Energy Technology published by Wiley-VCH GmbH **c** Solubility test of FAPbI_3_ dissolved in EtOH, EtOH/DMA mixed solvent, and EtOH/DMA solution with PACl **d** FTIR spectra of the precursor solutions. Characteristic peaks between 1620 and 1635 cm^− 1^ are assigned to the C = O stretching peaks of DMA. **e** Change of perovskite films with containing different types of RNH_3_Cl. Reprinted with permission from [37], Copyright 2022, The Author(s), under exclusive license to Springer Nature Limited. **f** Schematic of perovskite crystal formation in 2-ME-CHP solution system. Reprinted with permission from [39], Copyright 2021, Elsevier. **g** SEM images of perovskite film change according to the amount of NMP additives. Reprinted with permission from [38], Copyright 2022, American Chemical Society

Triethyl phosphate (TEP) is another promising greener solvent candidate to replace DMF. Recently, Cao et al. [30] provided a new approach to preparing uniform perovskite films using TEP as the host solvent. They prepared a composition of (FAPbI_3_)_1−x_(MAPbBr_3_)_x_ (x = 0, 0.05) perovskite for film formation. The solution was spin-coated and dibutyl ether, the low-toxic antisolvent, was dripped. Then, the P = O bond of TEP and the perovskite precursor immediately formed an intermediate phase, and the solvent complex was then converted to the perovskite phase through annealing treatment. The Fourier Transform Infrared Spectroscopy (FTIR) results in Fig. 4 explain this process well. The downshifted characteristic peak of the P = O bond supports the fact that a PbI_2_-FAI-TEP intermediate solvent complex was formed (see Fig. 3c). Finally, PCEs) of 20.13% (x = 0.05) and 18.65% (x = 0) were obtained based on the device configuration of FTO/SnO_2_/Perovskite/Spiro-OMeTAD/Au. Similarly, in 2023, Wu et al. [36] utilized this process to prepare a high-quality formamidinium (FA)-based perovskite from a TEP solvent. Dibutyl ether was used as an antisolvent, but the biggest difference from previous studies is that nanostructured tin oxide nanorods (SnO_2_-NRs) were used as an electron-transporting layer. They reported that the SnO2-NRs acted as a heterogeneous nucleation site for perovskite crystal formation, accelerating perovskite grain formation and inducing uniform grain growth (see Fig. 3d). Furthermore, a chlorine-terminated bifunctional supramolecule (Cl-BSM) was introduced to passivate the interfacial defects of the SnO_2_-NRs layer. As a result, it enabled a PCE of 22.42% based on the device configuration of FTO/SnO_2_-NRs/Perovskite/Spiro-OMeTAD/Ag with improved photo and humidity stability.

### DMSO

(DMSO) is a safe and environmentally friendly solvent that is commonly used as a co-solvent with DMF) in the fabrication of PSCs). However, using DMSO-based solutions alone for perovskite layer deposition has been difficult, due to the dewetting problem [18, 42] and complex quenching process on the underlying layer [43]. In 2021, Küffner et al. [32] addressed this issue by using a one-step blade coating method for inverted (p-i-n) PSCs with a DMSO-based solution at low processing temperatures. To prevent dewetting, they applied a blade-coated SiO_2_ nanoparticle (NP) wetting agent at the hole transport layer (HTL)/perovskite interface (see Fig. 4a). This resulted in a fully covered and homogeneous perovskite layer when deposited on the SiO_2_ NP layer. The resulting film exhibited a performance similar to that of the conventional DMF/DMSO (4:1 vol ratio) system. The fabricated double-cation PSCs showed an efficiency of 16.7% with a structure of indium tin oxide (ITO)/poly(triaryl amine) (PTAA)/SiO_2_ NPs/Perovskite/phenyl-C61-butyric acid methyl ester (PCBM)/bathocuproine (BCP)/Ag.

### GVL (GBL)

GBL have been used as an main solvent for perovskite precursors at very early stage works of PSCs [8]. Sufficient solubility of perovskite provided by GBL ensures a complete dissolution of perovskite, and it can be easily washed by dripping of antisolvent, such as toluene. Mixing of GBL, acetic acid and ethanol is also reported as an eco-friendly solvent system for perovskite layer, which exhibits similar performance with conventional solvent system [44]. However, GBL is a precursor of gamma-hydroxybutylic acid (GHB), which is a psychoactive drug that is prohibited seriously for usage worldwide. As an alternative, GVL is also used as a main solvent.

Worsley et al. [34] investigated γGVL) as a potential replacement for γGBL), which has issues with toxicity and legality. They suggested that GVL more readily coordinates to the Pb^2+^ center than GBL and thus forms smaller colloids. The UV-vis spectra result in Fig. 4b supports their assertions well. For cell fabrication, the precursor solution was drop-casted to form a stable mesoscopic carbon-based PSC, and was annealed at 50℃. The performance and stability were further improved by adding 5-aminovaleric acid (AVAI) to the precursor solution. The resulting cell, with a configuration of FTO/c-TiO_2_/mp-TiO_2_/m-ZrO_2_/m-carbon/AVA_0.03_MAPbI_3_, showed an efficiency of 12.91%, which is comparable to cells fabricated using GBL. They also found that the efficiency could be improved to 13.82% by adding methanol to the precursor solution with the same cell structure.

### EtOH

Yun et al. [37] recently proposed a novel solution process strategy, depositing high-quality perovskite films using an ethanol (EtOH) based solvent. Although many attempts have been made to produce films using EtOH, the solubility issue of PbI_2_ has been a significant obstacle. To address this problem, they prepared a stable perovskite precursor solution by adding dimethylacetamide (DMA) and alkylammonium chloride (RNH_3_Cl), resulting in a completely dissolved perovskite precursor solution. The PbI_2_ and FAPbI_3_ are insoluble in a pure EtOH or EtOH/DMA solution, but the addition of RNH_3_Cl enabled the formation of a soluble PbI_2_-HCl complex (see Fig. 4c). The shifted C = O peak in Fig. 4d, also confirms that FAPbI_3_ forms a strong Lewis acid-base interaction with DMA and PACl (n-pentylammonium chloride). Furthermore, the film’s morphology and electrical properties were investigated by varying the length of the alkyl group of the alkylammonium chloride. The researchers found that short alkylammonium chloride resulted in a low PCE) due to direct recombination from poor coverage, while long-chain ammonium chloride resulted in small grain size, leading to an increase in trap sites at the interface. The highest efficiency was achieved when n-butylammonium chloride was used. The optimized FTO/SnO_2_/Perovskite/Spiro-OMeTAD/Au structure achieved an extremely high efficiency of 25.08%.

### 2-ME

(2-ME) is one of the most suitable solvents for large-area coating, because of its low boiling point and high vapor pressure. Yoo et al. [39] utilized a precursor solution dissolved in 2-ME to fabricate a perovskite solar mini-module using an air-knife-assisted bar-coating method. Instead of the commonly used MAPbI_3_ perovskite composition in 2-ME-based solutions, they employed a (FAPbI_3_)_0.95_(MAPbBr_3_)_0.05_ composition with higher stability and an appropriate bandgap. However, when using 2-ME as a solvent, heterogeneous nucleation occurs more rapidly than with DMF, due to its relatively weak solvent-solute coordination. Therefore, 1-cyclohexyl-2-pyrrolidone (CHP) was used as an additive in the precursor solution to stabilize the film and to retard perovskite crystal growth. It was reported that the added CHP forms an intermediate phase (see Fig. 4f) with the perovskite precursor and balances the nucleation growth to produce a high-quality perovskite film. As a result, an efficiency of 20.3% was obtained in a mini-module with the structure of FTO/bl-TiO_2_/mp-TiO_2_/Perovskite/Spiro-OMeTAD/Au (aperture area of 31 cm^2^). Similarly. in 2022, Lee et al. [38] compared various solvents as additives to improve perovskite film quality using 2-ME. They reported that when NMP) was added to 2-ME, the NMP formed an intermediate phase and induced appropriate nucleation and crystal growth rates for film fabrication. When 40 mol% of NMP was added (NMP-0.4, see Fig. 4g), a uniform film with the highest performance was formed, and an efficiency of 20.39% was achieved in a unit device of the ITO/MeO-2PACz/Perovskite/PC_60_BM/BCP/Ag structure. The derivatives of 2-ME such as 2-BE (?) is also used as a solvent, but the longer alkyl chain resulted in a dramatical drop of solubility for perovskite layer, which limit their uses without huge amount of co-solvent [45].

## Eco-friendly fabrication process for perovskite films

Because the formation of the perovskite layer should be done by controlled crystallization, the usage of antisolvent is inevitable to obtain high-quality films. The amount of antisolvent used to fabricate a perovskite layer is much larger than the amount of solvent, and consequently the harmful antisolvent has a more severe effect on humans and the environment. Therefore, eco-friendly fabrication processes for PSCs can be achieved by two different ways. The easier way is to substitute the antisolvent with an eco-friendly, and less volatile one to prevent fire risk. An alternative approach involves modulating the crystallization behavior of the perovskite to enable the formation of a high-quality perovskite layer without the need for an antisolvent.

### Green antisolvent

In the perovskite formation process, the antisolvent should have good miscibility with the main solvent, but should not dissolve the precursor itself. It is also necessary to avoid the complete extraction of co-solvents or additives during the dripping process to allow for the formation of precursor-additive intermediates, and to finally achieve a highly crystalline, pin-hole-free film. The parameters for the selection of antisolvent have not yet been established, so a systematic establishment of selection criteria is required. Various solvent parameters including the Kamlet-Taft solvent parameter, Gutmann acceptor number (AN), or Dimroth-Reichardt E_T_, or a combination of them can be the ultimate solution to set the criteria for choosing the antisolvent.

The environmental considerations for antisolvent also should be similarly applied to the solvent, while considering the issues mentioned previously. Various solvents have been reported to be eco-friendly antisolvents. Ester-based solvents such as ethyl acetate (EA) and butyl acetate (BA), and alcohol-based solvents such as 2-propanol (IPA), n-butyl alcohol (n-BuOH), tert-butyl alcohol (t-BuOH), or anisole have been reported. Ethers are also frequently used as an antisolvent, but they have a very high risk of flammability [46]. Consideration of both aspects—selection of antisolvent based on interaction parameters and eco-friendliness of antisolvent itself—is of importance, but only few research has made effort to achieve this goal. Kim et al., suggested new eco-friendly antisolvent, t-BuOH, based on a consideration of solvent interactions by Dimroth-Reichard parameter, E_T_, and successfully demonstrate large-area film fabrication by antisolvent bathing, especially for pilot-scale roll-to-roll process [19]. The recent reports on eco-friendly antisolvent are summarized in Table 3.

**Table 3:** Recent reports of green-antisolvent-based fabrication for perovskite films

Antisolvent	Antisolvent additive	Structure	Precursor solvent	PCE%	References
IPA	–	ITO/NiO_x_/PTAA/MA_0.6_FA_0.4_PbI_3_/PCBM/BCP/Ag	DMF, DMSO	21.50	[47]
IPA	CTAC	ITO/SnO_2_/(FAPbI_3_)_0.92_(MAPbBr_3_)_0.08_/spiro-OMeTAD/Au	DMF, DMSO	23.4	[48]
isobutanol		ITO/TiO_2_/Cs_0.15_FA_0.85_PbI_3_/spiro-OMeTAD/Au	DMF, DMSO	22.03	[49]
EA	-	ITO/TiO_2_/MAPbI_3_/spiro-OMeTAD/Au	DMF, DMSO	17.3	[50]
EA	Acetylacetone	FTO/TiO_2_/MAPbI_3_/spiro-OMeTAD/Au	DMF, DMSO	21.1	[51]
diethyl carbonate	-	ITO/SnO_2_/PCBM/MAPbI_3_/spiro-OMeTAD/Ag	DMF, DMSO	20.20	[52]
(R)- (+)-limonene, 2-MeTHF		ITO/NiO_x_/MAPbI_3_/PCBM/Bis-C_60_/Ag	DMF, DMSO	18.04	[53]

#### IPA

**Fig. 5:**
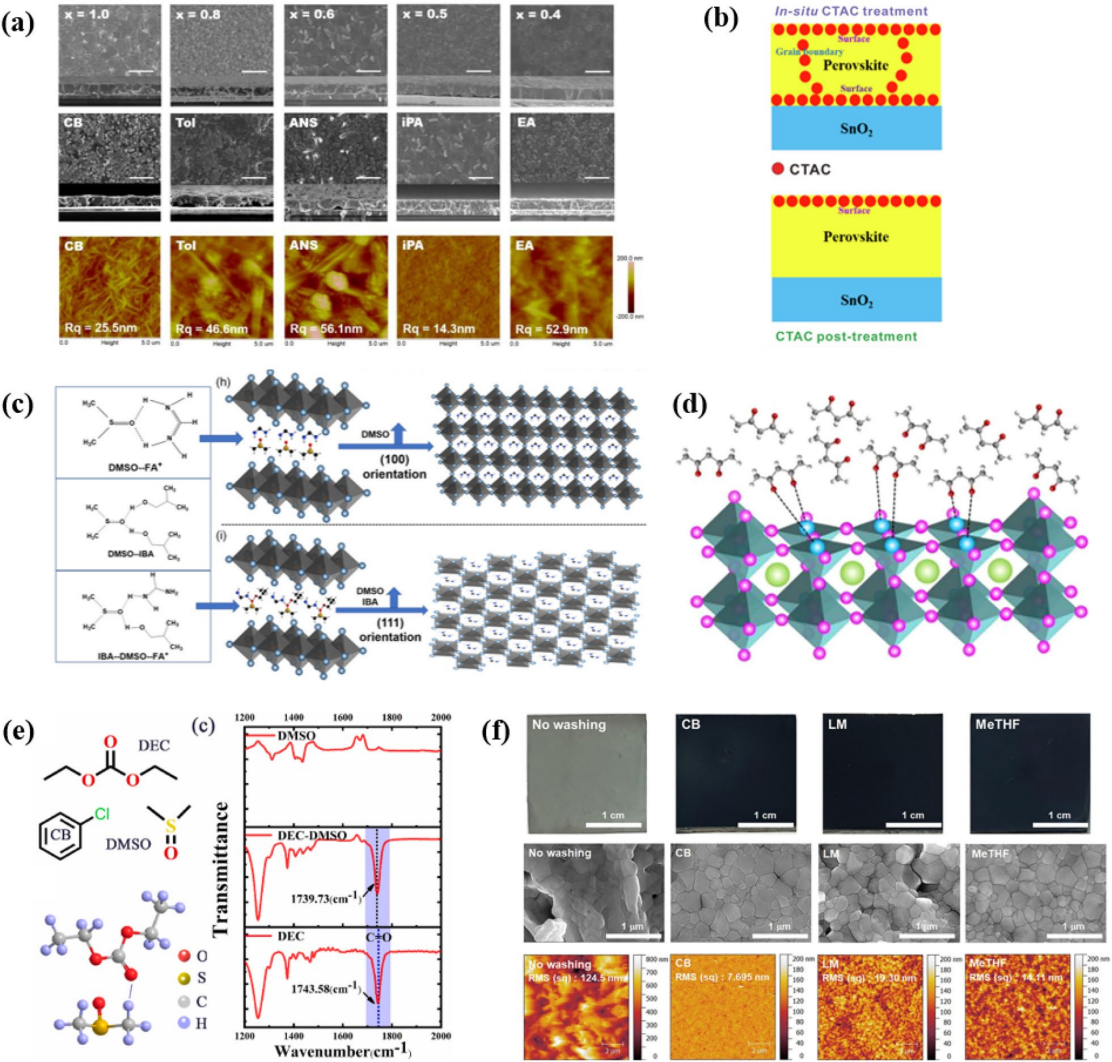
**a** Film morphologies depending on antisolvents. Reprinted with permission from [47], Copyright 2021, The Authors. *SusMat* published by Sichuan University and John Wiley & Sons Australia, Ltd. **b** Illustration of in-situ and post- CTAC treatment. Reprinted with permission from [48], Copyright 2021, Science China Press. **c** Illustration depicting the interaction between DMSO and FA^+^, and the preferred (111) crystal orientation. Reprinted with permission from [49], Copyright 2022, Wiley-VCH GmbH **d** Interaction between the C = O functional groups of acetylacetone and PbI_2_. Reprinted with permission from [51], Copyright 2021, Science Press and Dalian Institute of Chemical Physics, Chinese Academy of Sciences. **e** Illustration of hydrogen bond between a C= O group of diethyl carbonate and DMSO and FTIR spectrum presenting interaction between them. Reprinted with permission from [52], Copyright 2022, Elsevier B.V. **f** Uniform and pinhole-free perovskite films, made via green solvents. Reprinted with permission from [53], Copyright 2021, Elsevier B.V

Alcohols are the primary choice as green solvent except water in general. As an antisolvent, alcohols have been used frequently, despite the risk of dissolving a small amount of lead halide and selective dissolution of organic halide precursor. IPA, sec-butanol [54] or iso-butanol [55] have been used as an antisolvent. Shan et al. [47] utilized IPA as an antisolvent for perovskite film formation. The morphologies of perovskite films depending on the type of antisolvents were investigated (Fig. 5a). They determined the effect of the antisolvent on the intermediate phase. The appropriate combination of composition and antisolvent led to a PCE of 21.5%. Furthermore, they fabricated “fully green” devices with a PCE of 19.5%. For the perovskite layer, they excluded DMF and solely used DMSO as the solvent.

Moreover, Liu et al. [48] employed cetyltrimethylammonium (CTAC) along with IPA. The cetyltrimethylammonium and chloride groups heal the cation defects and anion vacancies, respectively. When CTAC was used as a post-treatment agent after the perovskite film formation, it only passivated the surface of the films. On the other hand, in situ treatment makes it possible to passivate both the surface and grain boundary defects (Fig. 5b). Due to the bifunctional action of CTAC, the PCE of the device reached 23.4%. This in-situ treatment also improved stability, allowing the device to retain 85% of its original efficiency after 600 h under illumination.

#### Isobutanol

Isobutanol, another type of alcohol, can be used as an antisolvent to achieve perovskite with superior crystal quality. Wang et al. [49] showed better crystallinity and preferred (111) crystal orientation by introducing isobutanol, which induces an interaction between DMSO and FA^+^ (Fig. 5c). This method showed a similar tendency in several compositions, demonstrating its universality. Among them, a Cs_0.15_FA_0.85_PbI_3_-based solar cell recorded a PCE of 22.03%.

#### EA

Other than alcohols, esters fall into another category of green solvents. A representative one is EA, which have been widely reported as an antisolvent. Other esters with different length of alkyl chains also have been reported [56]. EA has known to allow the perovskite crystallization process under ambient conditions. For instance, Chen et al. [50] investigated processability under high relative humidity (50–60%) using EA as an antisolvent. The ambient-process films with halide additives showed good morphologies without pinholes.

In addition, Li et al. [51] added acetylacetone to the EA to further enhance film quality. Acetylacetone in EA can permeate into perovskite wet film, forming an interaction between the C = O functional groups and PbI_2_ (Fig. 5d). Lone pair electrons from the O in C = O can restrain the generation of metallic lead, making the film more stable. The addition of acetylacetone showed a higher PCE of 21.1% than the 19.2% pristine.

#### Diethyl carbonate

Zhang et al. [52] reported the use of diethyl carbonate as a green solvent of CH_3_NH_3_PbI_3_ film. In contrast to chlorobenzene, a popular antisolvent, diethyl carbonate has a C = O group, and is able to hydrogen bond with DMSO (Fig. 5e). Interaction between diethyl carbonate and DMSO can assist the extraction of DMSO during the annealing process, giving large and compact grains. Superior perovskite films provided a decent PCE of 20.20%.

#### limonene & 2-MeTHF

Park et al. [53] employed (R)-(+)-limonene and 2-methyl tetrahydrofuran (2-MeTHF) as non-halogenated, non-aromatic green antisolvents. In addition, these two solvents can be obtained from bio-based processes. They claimed alcohol-based antisolvents were green, but their slight polarity is unsuitable for large-area processing. Uniform and pinhole-free perovskite films were made via these green solvents (Fig. 5f), and exhibited comparable performance with typical chlorobenzene-based ones. Moreover, for the PC_61_BM layer, they switched the solvent from chlorobenzene to o-xylene to make it close to an eco-friendly process.

### Antisolvent-free fabrication with use of DMF

Excluding the use of an antisolvent is another effective way to minimize the environmental hazards caused by the antisolvent. To achieve this goal, many attempts have been made to induce a high degree of supersaturation state by instantaneously increasing solubility by various methods, normally induced by antisolvent dripping.

**Fig. 6:**
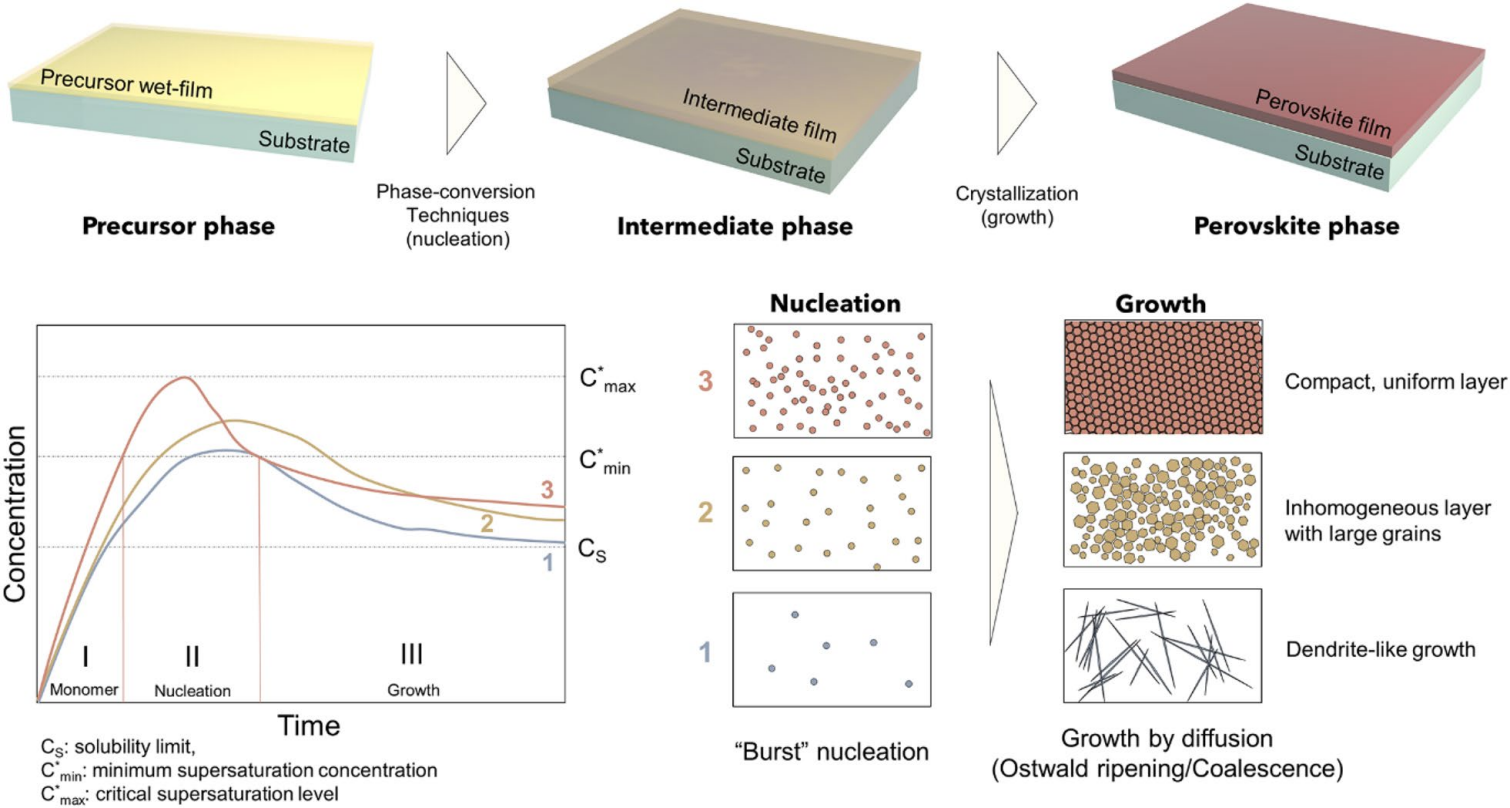
Schematic representation of perovskite film formation processes, and La Mer diagram with corresponding illustration of film morphologies for different cases. The degree of supersaturation level determines the number of seeds formed by “burst” nucleation and final morphology of the film after growth stage

As shown in Fig. 6, when the perovskite wet film is in contact with an antisolvent, the monomer concentration exceeds the solubility limit (Cs) and enters the supersaturation regime with a rapid decrease in solubility. (C^*^_min_ – C^*^_max_) Initial “burst” nucleation forms multiple “seeds”, and film is formed by growth step by diffusion of monomers according to different two mechanisms, Ostwald ripening or coalescence. The uniformity and density of the resulting thin film is depending on how quickly the monomer concentration increases, to what degree of supersaturation. A more rapid increase in monomer concentration, to a higher degree of supersaturation, results in the formation of more seeds with a uniform distribution in size, which eventually leads to a dense, high-quality film, predicted by the classical LaMer model. (Line 1–3 in Fig. 6, their consequences depend on the first nucleation stage)

To achieve this goal without using an antisolvent, various alternative methods have been previously reported. One approach to mimic this crystallization through solubility drop is the instantaneous removal of the main solvent by physical means, such as gas blowing or evacuating a vacuum, or to induce an instantaneous solubility difference during heat treatment through a combination of solvents with different solubilities and boiling points. Flash-infrared annealing, [57] vacuum flash, [58] N_2_ blowing, [39] and hot-pressing have been reported, which use different physical forces to remove the main solvent quickly. The use of a volatile main solvent with a strongly binding co-solvent, such as 2-ME:CHP), is also reported to induce the instantaneous formation of pseudo-intermediate phases, similar to the intermediate phase obtained by antisolvent dripping. The use of a high boiling point additive is another option, which effectively prevents rapid crystallization via strong binding between the additives and precursors. The utilization of additives or solvent combinations should be more cost-effective than the methods using physical means, but subtle control of the processing conditions may be required, and scalability is not guaranteed.

Even if the perovskite layer is successfully formed without an antisolvent, there are still health and environmental risks from the main solvent. Forming a perovskite layer using an environmentally friendly solvent without an antisolvent is the ultimate destination to reach. We have summarized the previous reports on the antisolvent-free fabrication of perovskite layer and classified them based on whether DMF is used or not as a main solvent, in Table 4.

**Table 4:** Recent reports of antisolvent-free fabrication for perovskite films

Method	Precursor solvent	Additive	Structure	PCE%	References
Vacuum + spin coating	DMF, DMSO		FTO/TiO_2_/Cs_0.15_FA_0.85_Pb(I_0.7_Br_0.3_)/GuaBr/spiro-OMeTAD/Au	19.67	[59]
Vacuum + blade coating	DMF, NMP	4-guanidinobutanoic acid	PEN/ITO/PTAA/FA_0.7_MA_0.25_Cs_0.05_Pb(I_0.93_Br_0.07_)_3_/PCBM/BCP/Ag	21.45	[60]
Spin coating	DMF, DMSO	ABTPbI_3_, MAPbCl_3_ microcrystals	ITO/MeO-2PACz/Cs_0.1_FA_0.9_PbI_3_/C_60_/BCP/Cu	23.27	[61]
Inkjet printing	DMF	PbAc_2_	ITO/ PEDOT:PSS/MAPbI_x_Cl_3-x_/PCBM/BCP/Ag	16.6	[47]
Gravure printing	DMF, DMSO	starch	PEN/ITO/SnO_2_/MAPbI_3_/P3HT/Au	~ 10	[62]

#### **Vacuum**

**Fig. 7:**
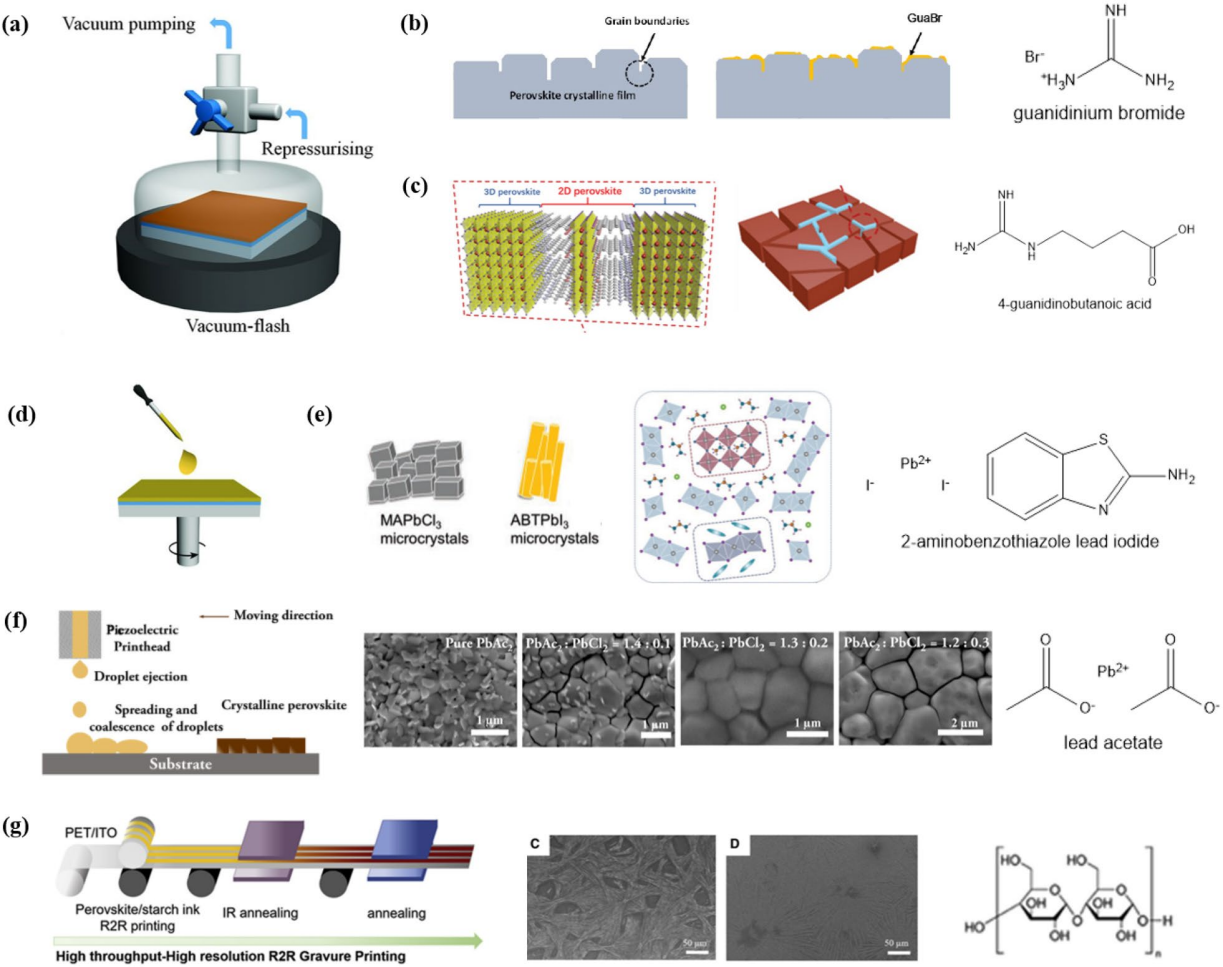
Schematic illustration of **a** vacuum-assisted solution processing, and **d** spin coating. Reprinted with permission from [58], Copyright 2016, American Association for the Advancement of Science. **b** Illustration showing surface treatment of vacuum-assisted perovskite films. Reprinted with permission from [59], Copyright 2021, Royal Society of Chemistry. **c** Illustration of 4-guanidinobutanoic acid forming 2D perovskites at the grain boundaries. Reprinted with permission from [60], Copyright 2021, The Authors. Advanced Science published by Wiley-VCH GmbH **e** Pre-synthesized 3D MAPbCl_3_ and 1D ABTPbI_3_) microcrystals as seed crystals during the film formation. Reprinted with permission from [61], Copyright 2022, Wiley-VCH GmbH **f** Schematic illustration of inkjet printing perovskite films and SEM images depicting film morphologies given by the balance between amounts of PbAc_2_ and PbCl_2_. Reprinted with permission from [47], Copyright 2021, Wiley-VCH GmbH **g** Schematic illustration of gravure printing process and the resulting films. Reprinted with permission from [62], Copyright 2021, The Author(s), published by Elsevier

Vacuum-assisted solution processing (VASP) is utilized to promote the homogeneous nucleation and uniform crystal growth of perovskite thin films (Fig. 7a). Rapid removal of relatively volatile main solvent via vacuum evacuation can effectively induce intermediate phase to be crystallized, and can be applied to large area [58]. Xia et al. [59] tested this methodology on a set of compositions with a range of bandgap values. (Fig. 7b) Blade coating is more suitable than spin coating for a scalable approach. Here, Wang et al. [60] applied blade coating to deposit a perovskite film with the assistance of a vacuum process. They determined that with vacuum-assisted blade coating fabrication, the widely used DMF:DMSO mixed solvent is inadequate. Since NMP has a stronger interaction with FA^+^ than DMSO, crystallization can become slower when a DMF:NMP mixed solution is adopted. As a result, in conjunction with 4-guanidinobutanoic acid as an additive (Fig. 7c), the PCEs have reached 21.45% and 20.16% on rigid and flexible substrates, respectively.

#### Spin coating

 Wang et al. [61] fabricated perovskite film by spin coating without using antisolvents. They pre-synthesized 3D MAPbCl_3_ and 1D 2-amino benzothiazole lead iodide (ABTPbI_3_) microcrystals, which can act as seed crystals (Fig. 7e). Microcrystals improved nucleation and crystal growth, facilitating the self-drying of the perovskite wet film. The un-modified perovskite wet film exhibited poor coverage with pinholes. On the other hand, the microcrystal-modified films became dense and uniform. Besides, the ABTPbI_3_ served as a grain boundary passivator, contributing to the PCE enhancement, reaching 23.27%.

#### Inkjet printing

Inkjet printing is adequate for large-area patterning. However, most inkjet printing techniques form thick, wet films. These solvent-rich layers are not suitable to forming uniform and compact crystalline films. Zhang et al. [47] developed an inkjet printing process with picoliter-sized droplets to remove the costly vacuum-assisted annealing stage. The mixture of two salts, lead acetate and lead chloride, were used for the perovskite. During the reaction process, PbAc_2_ is converted to MAAc, which is more volatile than methylammonium chloride (MACl). The balance between the amounts of PbAc_2_ and PbCl_2_ gave the favored crystal structure (Fig. 7f). With the 1.6 μm-thick perovskite film, a PCE of 16.6% was recorded.

#### Gravure printing

 Antisolvent bathing is generally needed for roll-to-roll gravure-printed perovskite films, which is proven by previous successful demonstration of PSCs [19, 20]. However, bathing takes up one more stage in the manufacturing process, making it more costly and time-consuming. Bisconti et al. [62] introduced starch as a rheological modifier in perovskite ink. Starch can boost the viscosity of a precursor solution while maintaining a low concentration of solutes, making it appropriate for gravure printing. The viscosity of a 0.75 M solution with starch is comparable to a 2 M solution, minimizing the dissipation of precursor materials. Furthermore, the hydroxyl groups of starch interact with the MA^+^ and DMSO, delaying the crystallization process (Fig. 7g). In turn, uniform perovskite film has been made without antisolvents. This resulted in a PCE near 10%, under ambient conditions. 

### Antisolvent-free fabrication with no use of DMF

#### D-bar coating + N _2_ blowing, ACN

**Fig. 8:**
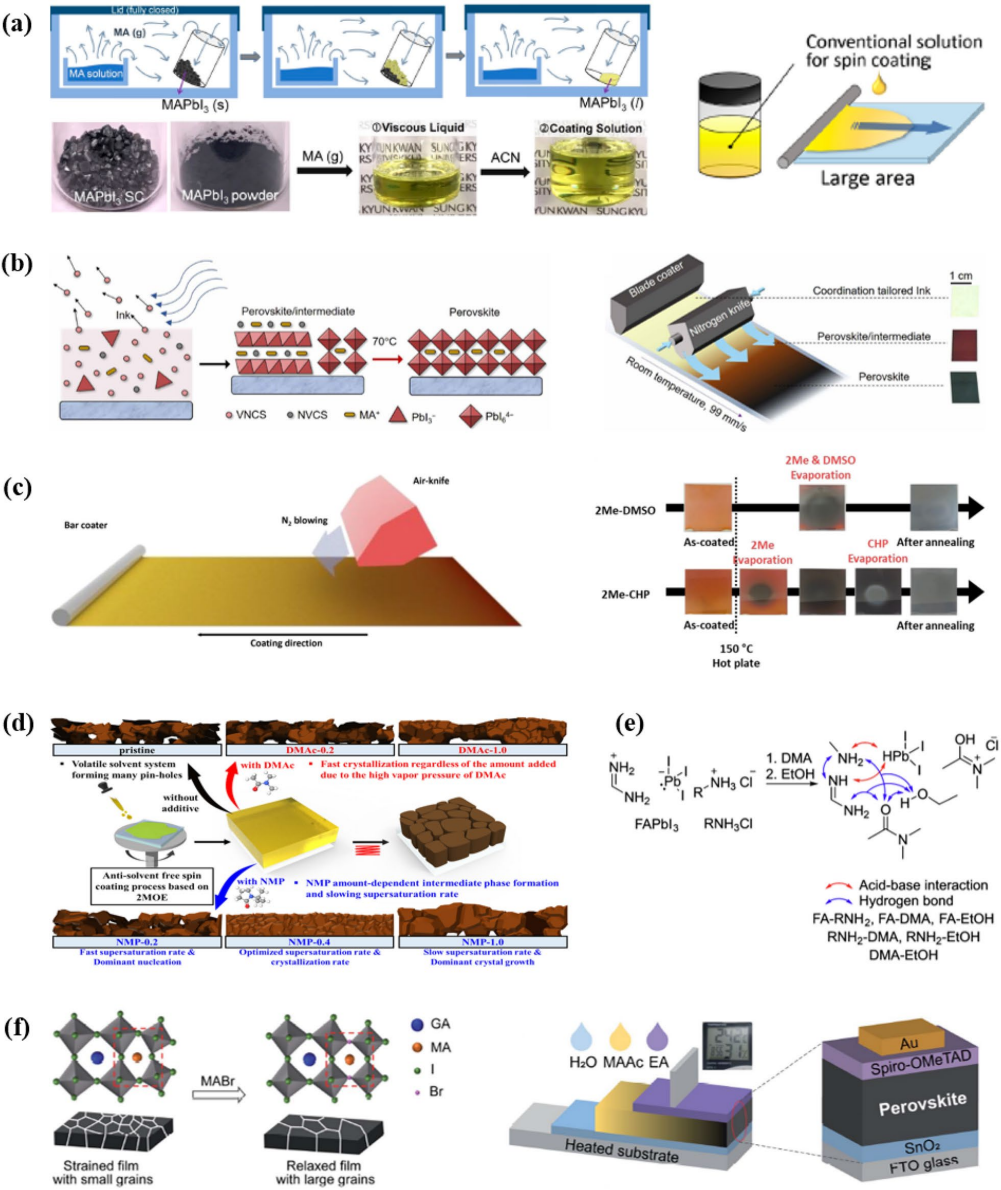
**a** Gas-mediated solid-liquid conversion process and D-bar coating of the solution. Reprinted with permission from [63], Copyright 2019, American Chemical Society. **b** The crystallization process of perovskite precursor solution employing ACN and 2-ME as the solvent and its rapid blading method. Reprinted with permission from [17], Copyright 2019, The Authors, some rights reserved; exclusive licensee American Association for the Advancement of Science. **c** Fabrication of perovskite films with 2-ME and CHP mixed solution. Reprinted with permission from [39], Copyright 2021, Elsevier Inc. **d** The role of NMP in precursor solution. Reprinted with permission from [38], Copyright 2022, American Chemical Society. **e** The addition of RNH_3_Cl, enabling the formation of a soluble PbI_2_-HCl complex. Reprinted with permission from [37], Copyright 2022, The Author(s), under exclusive license to Springer Nature Limited. **f** Effect of MABr, releasing residual lattice strain to stabilize the crystal structure. Device structure with green solvents used for each layer. Reprinted with permission from [64], Copyright 2021, Royal Society of Chemistry

 Gas blowing is another effective strategy to remove main solvent quickly, and induces uniform intermediate phases. Rather than directly dissolving precursors in solvents, an MAPbI_3_ solution can be prepared by gas-mediated solid-liquid conversion. MAPbI_3_ single crystals are placed together with the MA solution. As time passes, the black-colored powder turns into a yellowish liquid. Jeong et al. [63] used this liquid MAPbI_3_ for D-bar coating (Fig. 8a and Table 5). For effective coating, the solution should have low viscosity and a low boiling point for easy evaporation. Therefore, ACN was selected as a solvent to mix with the liquid MAPbI_3_. In other kinds of solvents, such as DMF and DMA, the film displayed worse morphologies.

**Table 5:** Reports on antisolvent-free & DMF-free fabrication for perovskite films

Precursor solvent	Additive	Method	Structure	PCE%	References
ACN	MA gas	D-bar coating + N_2_ blowing	FTO/SnO_2_/MAPbI_3_/spiro-OMeTAD/Au	17.82	[63]
ACN	MA gas	spin coating	ITO/E-ZnO/MAPbI_3_/spiro-OMeTAD/Au	20.39	[50]
ACN	MA gas	Solution shearing method	FTO/c-TiO_2_/mp-TiO_2_/MAPbI_3_/Spiro-OMeTAD/Ag	20.30	[29]
ACN, 2ME	DMSO	blade coating + N2 flow	ITO/PTAA/MAPbI_3_/C_60_/BCP/electrode	16.4	[17]
EtOH, DMA	RNH_3_Cl	spin coating	FTO/SnO_2_/FAPbI_3_/Spiro-OMeTAD/Au	25.1	[37]
2ME	NMP	spin coating	ITO/MeO-2PACz/MAPbI_3-x_Cl_x_/PC_60_BM/BCP/Ag	20.39	[38]
2ME	CHP	Air-knife assisted bar-coating	FTO/bl-TiO_2_/mp-TiO_2_/(FAPbI_3_)_0.95_(MAPbBr_3_)_0.05_/Spiro-OMeTAD/Au	20.3	[39]
MAAc	MABr	blade coating	FTO/SnO_2_/GA_0.12_MA_0.88_PbI_3_/spiro-OMeTAD/Au	20.21	[64]

#### Spin coating, ACN

 Wang et al. [50] also used this MAPbI_3_(*l*)-ACN solution for film processing. When employing ZnO as an electron-selective layer, annealing can accelerate the decomposition of the superjacent perovskite layer. To overcome this hurdle, they explored a perovskite film fabrication that does not require an annealing and antisolvent process. By spin-coating the MAPbI_3_-ACN solution, perovskite film was instantly formed. Accompanied by ZnO modification, a PCE of 20.39% was obtained.

#### Blade coating, ACN + 2ME

Deng et al. [17] employed a solvent mixture of ACN and 2ME for the rapid blade coating of perovskite films. The simultaneous use of volatile noncoordinating solvent and nonvolatile coordination solvent makes it possible to obtain fine-quality films within a short processing time. The small amount of DMSO, a nonvolatile coordination solvent, aids the coordination ability until it evaporates, making the grain size larger (Fig. 8b). The device consisting of perovskite film fabricated under an ambient atmosphere with a blading speed of 99 mm/s recorded a PCE of 16.4%.

#### Blade coating, MAAcs

Excluding the antisolvent while fabricating perovskite makes it tough to obtain uniform films. Fang et al. [64] employed methylammonium acetate (MAAc) as the green solvent for precursor ink and controlled the wetting and spreading of the viscous solution. Furthermore, MABr was used to release residual lattice strain. Since GA^+^ ions are larger in size than MA^+^ or FA^+^, it makes the lattice expand. MABr was added to the fundamental composition (GA_0.12_MA_0.88_PbI_3_) to make the lattice structure stable (Fig. 8f). Consequently, films with large grains have resulted. In addition, they also fabricated charge transport layers with green solvents. Through these approaches, they achieved a PCE of 20.21%.

## Conclusions

In conclusion, we have discussed the recent advancements in environmentally friendly fabrication methods for producing highly efficient PSCs, as well as the latest developments in green solvent systems. The drive towards large-scale production of perovskite solar modules has caught the attention of many researchers in the global photovoltaic community, particularly in industrial areas. In order to achieve commercial feasibility, several key factors must be taken into consideration when transitioning from lab-scale to pilot-scale production. These include assessing the potential health risks posed by the processing solvents used in perovskite ink, identifying the most suitable fabrication processes for achieving uniform, pin-hole-free large-area perovskite layers, and developing scalable coating technologies for antisolvent-free fabrication processes that employ stable perovskite inks and green solvents. To find the answer, our review focused on studies that explored two key aspects: (1) the design and preparation of perovskite ink using green solvents, and (2) scale-up fabrication methods which use no antisolvents.

To begin with, it is crucial to understand the process by which high-quality polycrystalline perovskite films are formed in solution-processable fabrication, which involves controlling factors such as coating speed, solvent evaporation rate, nucleation and growth rate, and thermal annealing. The commonly used perovskite ink solvents are DMF/DMSO co-solvents, where DMF acts as the main solvent and DMSO serves as a co-solvent or mediator. However, in order to eliminate the use of toxic DMF, various attempts have been made to employ less toxic or eco-friendly solvents such as EtOH, ACN, GVL(GBL), TEP, and others. Nevertheless, the use of a co-solvent or mediator with a high coordinating affinity with PbI_2_ is still required to dissolve the perovskite precursors.

For instance, DMSO is an aprotic polar solvent and a Lewis base that can coordinate with Lewis acid, PbI_2_, facilitating the dissolution of the perovskite precursors in its mixture with alternatives to DMF. In the solvent engineering method, the role of DMSO as a co-solvent or mediator is to slow down the rapid crystallization of the perovskite precursors during the wet film solidification. After depositing the perovskite ink, the concentration of the precursors in the solution increases as the solvents evaporate. The excess solvents are then washed away from the wet film by solvent extraction via anti-solvent dripping or bathing, resulting in a rapid formation of a supersaturated state of the perovskite precursors in the film. At this point, the nucleation and growth process occur, where numerous small crystals are generated through nucleation, followed by gradual growth into larger crystals to finally form a dense and uniform perovskite film with full surface coverage.

In antisolvent dripping or bathing methods, toluene, chlorobenzene, and diethyl ether were commonly employed in the past, but they have been recently replaced with green solvents such as IPA, isobutanol, EA, diethyl carbonate, 2Me-THF, and others. However, when an antisolvent is used, a large amount of antisolvent waste, ventilation and recycling facilities must be necessarily considered, restricting the commercialization of PSCs. Thus, the ultimate goal is to develop antisolvent-free fabrication methods.

In order to achieve a dense and uniform perovskite thin film, it is necessary to rapidly create a supersaturated state in the perovskite precursors within the film without the use of an antisolvent. According to the La Mer mechanism, the formation of a burst (or rapid) nucleation is essential for generating uniform small crystals (or seeds). This can be induced through several methods, such as increasing the substrate temperature, rapidly blowing gas, applying vacuum, and using highly volatile solvents, which are similar to the use of antisolvent. To this end, several methods such as air or nitrogen blowing-assisted bar (or blade) coating, vacuum-assisted blade coating, picoliter-sized droplets-used ink-jet printing, and high-viscosity ink-used gravure printing have been successfully demonstrated.

However, implementing these methods alone is insufficient to ensure the formation of a dense and pinhole-free perovskite film. To fabricate a highly crystalline film with full surface coverage, it is necessary to carefully design and prepare the perovskite ink, consisting of a main solvent, a co-solvent or mediator, and additives. Co-solvents or mediators such as DMSO, DMAc, NMP, and CHP can be utilized in a mixture with the main solvent, due to their higher boiling points and lower vapor pressures, which facilitate continuous network growth of microstructures in the perovskite film.

DMAc, NMP, and CHP are classified as hazardous to health and environment, with DMSO the lowest. MA gas has been used to dissolve the MAPbI_3_ through gas-mediated solid-liquid conversion. Here, methylammonium acetate serves as the sole solvent.

Proper additives are also essential to improve the crystallinity and surface morphology of the perovskite layer. Ammonium chloride (NH_4_Cl) and MACl) are typically used to determine the phase-conversion rate from the delta (hexagonal) to alpha (cubic), preferred crystal orientation, and the grain size of alpha-phase FA-based perovskites. Recently, propyl and butylammonium chloride have also been used in combination with MACl due to their slower volatilization rates. These additives are converted into volatile species by decomposition and are evaporated during a thermal annealing step.

On the other hand, CTAC) and 4-guanidinobutanoic acid are added to the precursor ink and those additives remain in the perovskite layer to reduce trap defects. Finally, using starch as a polymer in the precursor solution can enhance the ink’s viscosity for printing processes such as gravure printing.

Future work is also required to develop a perovskite ink that is tolerant of the pilot-scale fabrication environment in order to accelerate the commercialization of PSCs. The majority of effective PSCs utilize DMSO as a co-solvent or mediator. However, the use of DMSO in the perovskite ink does not guarantee a high level of reproducibility and a sufficient processing window when the film is fabricated in a less-controlled environment with a wide range of relative humidity and temperature. There are only a few reports that meet both the environmentally friendly and tolerant fabrication requirements in which methylamine formate and methylsulfonylmethane were used instead of DMSO [65, 66].

In this review, we have systematically discussed recent advances in eco-friendly manufacturing processes for PSCs), focusing on two key issues: (1) the design and preparation of perovskite ink using green solvents, and (2) the development of fabrication methods without the use of antisolvents. Various attempts have been made to identify less toxic and environmentally friendly solvents for perovskite ink, as well as to develop antisolvent-free fabrication processes. Nevertheless, challenges still persist. It should enable newly developed eco-friendly solvent system to mimic the crystallization behavior of perovskite film made by traditional solvents and antisolvent dripping processes. The new solvent system should include the main solvent having sufficient solubility to perovskite precursor and co-solvent/mediator having suitable binding strength to the precursor. The deeper and systematic understanding of perovskite crystallization behavior, and establishment of selection criteria for solvents for perovskite precursors is necessary. Binding strength to perovskite precursor and volatility of solvents themselves should be carefully controlled, thereby inducing a high-quality, uniform intermediate phase only by suitable physical means. In addition, eco-friendliness should be assessed in consideration of various perspectives, not just by a single quantitative measure. In order to achieve an environmentally friendly and sustainable production process, it is necessary to consider not only the single use of solvents but also the harmfulness of raw materials and production processes for production of solvent, as well as the cost and environmental impact of solvent reuse after heat treatment or evaporation. The newly adopted technologies must not only guarantee the production of high-quality and uniform perovskite films, but also achieve this at a large-area scale and with low-cost production, which are indispensable requirements for the commercial-scale fabrication of PSCs.

## Data Availability

Not applicable.
